# P-1548. Identification of novel shared antibodies and infectious disease associations in Alzheimer’s disease

**DOI:** 10.1093/ofid/ofaf695.1728

**Published:** 2026-01-11

**Authors:** Arthur Chang, Kinga Szigeti, Mark D Hicar

**Affiliations:** University of Nebraska Medical Center, Elkhorn, Nebraska; University at Buffalo, Buffalo, New York; University at Buffalo, Buffalo, New York

## Abstract

**Background:**

Recent data support an infectious disease relationship with development of Alzheimer’s disease (AD). All infections will cause an adaptive immune response with both T cell and B cell clonality. The diversity of an antibody (Ab) is generally maximal in the complementarity determining region 3 (CDR3) which is transcribed from the region of joining of the sorted V, D, and J gene segments and is targeted by somatic hypermutation. Despite this potential for diversity, shared Ab sequences between individuals in response to the same infection have been described for HIV and SARS-CoV-2. These so-called “public clonotype” Abs (PCAbs) have been shown to be specific for the preceding infection. We hypothesize that PCAbs unique to AD will reveal an infectious disease related to initiation or contribution to AD.

**Figure:**
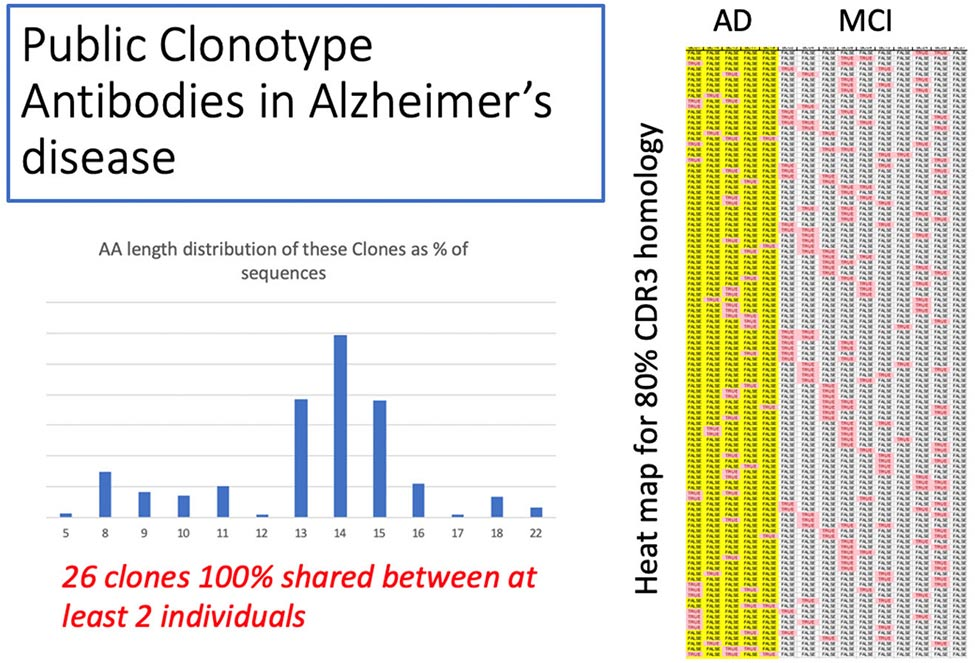
Public Clonotype Antibodies In Alzheimer's Disease The variable gene, d segment and joining segment, upon recombination in the B cell, contribute genetic material to the final antibody region termed the complementarity determining region three (CDR3). This makes the CDR3 region extremely diverse and the CDR3 tends to drive the most specific binding to foreign antigens. We used compared sequences using 100% and 80% amino acid CDR3 homology to describe antibodies shared amongst individuals with Alzheimer's or Mild Cognitive Impairment.

**Methods:**

PBMCs were isolated from AD, mild cognitive impairment (MCI) and relative age matched controls (spouses). Plasma VirScan (CDI laboratories) screens ( >400,000 phage displayed peptides, >200 common viruses) were performed. Memory B cells were separated from PBMCs using the Miltenyi Memory B Cell Isolation kit. Heavy chain variable gene sequences were sequenced (Irepertoire). Select samples were also single cell sequenced by using 10x genomics platform to provide pairing of heavy and light chain. Sequences were processed with Immcantation software. Homology was defined by use of the same predicted Variable gene and J segment and similar CDR3.

**Results:**

Virscan phage screening identified 46 peptides. Half were to a member of the Herpesviridae and 7 represented two epitopes in a single protein of a virus not formerly associated with AD (Agent X). Defining PCAbs as using the same predicted V gene and J segment and 80% amino acid identify of the specific CDR3 Ab region, a total of 770 PCAbs are specific to members of AD/MCI. For monoclonal Ab production, we prioritized Abs that showed evidence of antigen targeting: class-switching, high mutation rates from germline, and bias for amino acid replacement mutations. We utilized 10x genomics sequences to provide light chains for monoclonal Ab production and are producing 18 Abs for initial production.

**Conclusion:**

We have found a novel viral association with AD with ongoing studies assessing monoclonal Ab targeting to Agent X.

**Disclosures:**

Mark D. Hicar, MD/PhD, Pfizer: site-PI for vaccine study on Lyme

